# Association Between Texture-Modified Diet Intake Rate and Functional Outcomes After Acute Stroke: A Retrospective Observational Study

**DOI:** 10.3390/nu18193114

**Published:** 2026-09-22

**Authors:** Rie Motoyama, Tomotaka Tanaka, Kazuki Fukuma, Shinsaku Nakazawa, Tetsuya Chiba, Shuichi Tonomura, Masafumi Ihara

**Affiliations:** 1 Department of Neurology, National Cerebral and Cardiovascular Center, Suita 564-8565, Osaka, Japan; motoyama.rie@ncvc.go.jp (R.M.); kazuki.fukuma@ncvc.go.jp (K.F.); nakazawa.shinsaku@ncvc.go.jp (S.N.); chiba.tetsuya@ncvc.go.jp (T.C.); tonomura.shuichi58@ncvc.go.jp (S.T.); 2 Department of Neurology, Faculty of Medicine, Shimane University, Izumo 693-0021, Shimane, Japan

**Keywords:** stroke, dysphagia, oral intake, texture-modified diet

## Abstract

Background/Objectives: Texture-modified diets (TMDs) are commonly prescribed after stroke, but the clinical significance of the amount actually consumed remains unclear. We examined the association between TMD intake rate and functional status at discharge and 90 days after stroke. Methods: This retrospective single-center study included 406 patients with ischemic stroke or intracerebral hemorrhage who received TMDs for suspected or confirmed dysphagia between 2012 and 2017. Overall TMD intake was calculated as the meal-count-weighted mean consumption proportion across four TMD levels and analyzed per 10% increase. Ordinal logistic regression models for modified Rankin Scale (mRS) scores were adjusted for age, sex, admission National Institutes of Health Stroke Scale (NIHSS) score, premorbid mRS, stroke type, and admission Geriatric Nutritional Risk Index (GNRI). Binary logistic regression for a favorable outcome (mRS 0–2) was performed as a secondary analysis. Results: Each 10% increase in TMD intake was associated with lower odds of a higher mRS category at discharge (adjusted OR, 0.85; 95% CI, 0.77–0.94) and at 90 days (adjusted OR, 0.88; 95% CI, 0.80–0.96). Higher intake was associated with higher odds of a favorable outcome at discharge (adjusted OR, 1.31; 95% CI, 1.03–1.66), whereas the 90-day association was not statistically significant (adjusted OR, 1.15; 95% CI, 0.98–1.35). Diet-specific associations were inconsistent. Conclusions: Higher TMD intake rates were associated with better functional status after multivariable adjustment. Because TMD intake may also reflect swallowing impairment, clinical condition, and early recovery, these observational findings do not establish causality. Monitoring TMD intake may help identify patients who warrant closer nutritional and swallowing assessment.

## 1. Introduction

Stroke remains a leading cause of long-term disability, and functional outcomes after acute stroke are strongly influenced by baseline neurological severity [1]. However, in addition to neurological impairment, post-stroke complications and supportive care during the acute phase, including nutritional management, may also influence the recovery trajectory [2].

Dysphagia is one of the most clinically significant complications of stroke. It can lead to aspiration pneumonia and reduce oral intake, thereby increasing the risk of dehydration and undernutrition, both of which can interfere with early rehabilitation and functional recovery [3,4,5]. Previous studies have shown that post-stroke dysphagia is associated with malnutrition [6], and nutritional impairment is increasingly recognized as a potentially modifiable factor in post-stroke rehabilitation. Accordingly, nutritional management during the acute phase should be considered an integral component of comprehensive stroke care rather than merely supportive care. This is aligned with current clinical guidelines that emphasize early formal screening, dysphagia management, and structured rehabilitation to optimize functional recovery [7].

Texture-modified diets (TMDs) are commonly prescribed for patients with dysphagia to maintain safe oral intake while reducing the risk of aspiration [8,9]. These diets are designed to modify food texture, viscosity, and consistency according to patients’ swallowing ability, and the prescribed meal plan is generally intended to provide adequate energy and protein when consumed in full. Therefore, the nutritional benefit of TMDs depends not only on the appropriateness of the prescribed texture but also on the actual amount consumed. Recent evidence suggests that dietary modifications may improve energy and protein intake in adults with dysphagia, highlighting the importance of balancing swallowing safety with adequate nutritional provision [10]. Nevertheless, in routine clinical practice, patients may leave a considerable proportion of their meals uneaten because of impaired consciousness, fatigue, appetite loss, oral-phase dysfunction, cognitive impairment, or poor palatability of modified diets. Previous structured evaluations have shown that individuals prescribed standard hospital TMDs often fail to meet their estimated energy and protein requirements owing to a combination of factors including poor palatability, incorrect meal delivery, and low rates of meal consumption, highlighting the gap between prescribed nutrition and actual intake [11].

Several scales have been developed to evaluate oral intake in patients with dysphagia. For example, the Functional Oral Intake Scale (FOIS) is a validated ordinal scale that describes the functional level of oral intake after stroke [12]. However, these scales primarily assess the route or level of oral intake and do not directly quantify the amount of food consumed. From a nutritional and rehabilitation perspective, the proportion of meals consumed may provide additional clinically relevant information because it reflects actual oral intake and the extent to which prescribed nutritional provision is actually consumed in routine care. For example, fluid thickeners and texturizers are routinely prescribed to prevent aspiration, yet insufficient fluid intake and subsequent dehydration are frequently observed in patients with acute stroke and dysphagia [13,14].

Recent studies have examined food consumption as a prognostic nutritional parameter in patients after stroke. Siotto et al. [15] reported that malnutrition and food intake were associated with recovery of activities of daily living in patients undergoing subacute stroke rehabilitation. They also found that greater waste of the “second dish,” which mainly contained protein sources, was independently associated with poorer functional recovery after adjustment for age and serum albumin [15]. In addition, pre-existing sarcopenia in patients with acute stroke has been associated with impaired oral intake and aspiration pneumonia [16]. These findings suggest that reduced food intake after stroke may reflect not only swallowing dysfunction but also broader nutritional and muscle-related vulnerabilities. Collectively, this evidence supports the hypothesis that actual food intake, rather than diet prescription alone, may be clinically relevant to functional recovery after stroke. However, evidence regarding the association between the actual TMD intake rate and functional outcomes in patients with acute stroke remains limited. Therefore, we investigated whether the TMD intake rate was associated with modified Rankin Scale (mRS) scores at discharge and 90 days after stroke, after multivariable adjustment. We hypothesized that a higher TMD intake rate would be associated with more favorable functional outcomes.

## 2. Materials and Methods

### 2.1. Study Design and Participants

This retrospective observational study included patients with acute ischemic stroke or intracerebral hemorrhage who were admitted to the National Cerebral and Cardiovascular Center between January 2012 and December 2017. Eligible patients were those prescribed TMDs for suspected or confirmed dysphagia during hospitalization. TMDs were prescribed based on clinical swallowing assessments performed by the treating physicians and nurses. The exclusion criteria were as follows: (1) missing data for key variables (admission National Institutes of Health Stroke Scale (NIHSS) scores, diet records, or outcomes); (2) fewer than 5 consecutive days of TMD consumption.

### 2.2. Exposure: TMD Intake Rate

TMDs were categorized into four levels used in our institution: jelly diet, paste diet, mousse diet, and moist minced diet. Representative photographs of currently provided meals corresponding to the TMD categories examined in this study are shown in Supplementary Figure S1. These diet categories were institution-specific and predated implementation of the International Dysphagia Diet Standardisation Initiative (IDDSI) framework at our institution. Because the diets were not classified using standardized IDDSI testing methods during the study period, reliable one-to-one retrospective mapping to IDDSI levels was not possible. Swallowing function was initially assessed using the Modified Water Swallowing Test (MWST) at admission, and the initial TMD level was selected based on the test results. Thereafter, diet texture was dynamically adjusted according to the patient’s clinical tolerance during oral intake. The diet level was downgraded when swallowing-related problems, such as coughing or choking during meals, were observed, whereas it was upgraded when the patient was able to consume the current diet without apparent difficulty. Speech-language-hearing therapists also assessed swallowing function when clinically indicated and contributed to adjustment of the diet texture. Thus, the diet type prescribed to each patient could change during hospitalization according to swallowing status and clinical course. At each meal, nursing staff visually estimated and recorded the amount consumed separately for the staple foods and side dishes using a scale ranging from 0 to 10, with 10 indicating complete consumption, 5 indicating approximately half of the served portion, and 0 indicating no consumption. The recorded values were subsequently converted to proportions ranging from 0 to 1 (e.g., 10 = 1.0, 5 = 0.5, and 3 = 0.3). For each diet level, the proportions consumed across all corresponding meals were summed separately for the staple food and side dishes and divided by the number of meals at that diet level, yielding the mean intake rate for each component. The diet-level intake rate was then calculated as the arithmetic mean of the staple-food and side-dish intake rates. These two components were weighted equally because routine clinical records did not provide sufficiently detailed information on the absolute weight or nutrient content of each component for every meal to support differential weighting. The overall TMD intake rate was then calculated as the meal-count-weighted average across all diet levels, using the following formula:

Overall TMD intake rate = Σ (mean intake rate for each diet level × number of meals served at that diet level)/total number of TMD meals served.

For example, if a patient received a paste diet for 10 meals with a mean intake rate of 0.8 and a mousse diet for 5 meals with a mean intake rate of 0.6, the overall intake rate was calculated as follows:

(0.8 × 10 + 0.6 × 5)/15 = 0.73.

Thus, the overall TMD intake rate represented the meal-count-weighted mean proportion of prescribed TMDs consumed during the period of TMD provision. Only meals provided while TMDs were prescribed during hospitalization were included in the calculation. For the exploratory meal-component analysis, overall staple-food and side-dish intake rates were calculated separately as meal-count-weighted averages of the corresponding component-specific intake rates across all TMD levels. To facilitate clinical interpretation, all intake rates were analyzed per 10% increase.

### 2.3. Outcomes

The primary outcomes were functional status, assessed using the mRS at discharge and 90 days after stroke onset. The mRS is a 7-category ordinal scale ranging from 0 (no symptoms) to 6 (death), with higher scores indicating greater disability. The discharge mRS was assessed at the end of the same hospitalization during which TMD intake was recorded, whereas the 90-day mRS was assessed subsequently. The 90-day mRS score was obtained from outpatient follow-up visits, telephone interviews, or medical records from transfer hospitals. When the 90-day assessment was performed outside the index hospital, the available clinical information was reviewed to determine the mRS score. A favorable functional outcome, defined as an mRS score of 0–2, was analyzed as a secondary binary outcome.

### 2.4. Covariates

The following baseline variables were included as adjustment covariates in the primary multivariable models: age, sex, admission NIHSS score, premorbid mRS score, stroke type, and admission Geriatric Nutritional Risk Index (GNRI). These variables were selected based on their clinical relevance to functional outcomes and/or oral intake after stroke. The admission NIHSS score was included as a measure of baseline neurological severity, premorbid mRS as a measure of pre-stroke functional status, and GNRI as an indicator of baseline nutritional status. Stroke type was categorized as ischemic stroke or intracerebral hemorrhage.

### 2.5. Statistical Analysis

Continuous variables were presented as medians and interquartile ranges (IQRs), whereas categorical variables were presented as numbers and percentages. The primary association between the overall TMD intake rate and mRS scores was examined using multivariable ordinal logistic regression with a cumulative logit model. All primary models were adjusted for age, sex, admission NIHSS score, premorbid mRS score, stroke type, and admission GNRI. Effect sizes were reported as adjusted odds ratios (ORs) per 10% increase in the TMD intake rate with 95% confidence intervals (CIs). In this model, an OR < 1 indicated lower odds of being in a higher (worse) mRS category. The proportional odds assumption was assessed using the parallel lines test. Because the unconstrained model did not adequately converge for some analyses, particularly for the 90-day mRS outcome, the formal test could not be reliably interpreted. Therefore, for the 90-day mRS outcome, threshold-specific binary logistic regression models were additionally examined across all possible mRS cut points to assess the consistency of the direction of association. Secondary analyses were performed using binary logistic regression to evaluate favorable functional outcomes (mRS 0–2). These analyses were considered secondary because dichotomization of the mRS may reduce statistical information compared with ordinal analysis. As an additional sensitivity analysis, hypertension, dyslipidemia, diabetes mellitus, and atrial fibrillation were added to the primary multivariable models to assess the robustness of the findings to further adjustment for major comorbidities. Exploratory diet-specific analyses were also performed using multivariable ordinal logistic regression, with adjustment for the same covariates as in the primary models. Each diet-specific analysis included patients who had received the corresponding diet type during hospitalization. Because some patients received more than one diet type during hospitalization, the diet-specific patient groups were not mutually exclusive. Because diet type was selected and modified according to swallowing status and overall clinical condition, these analyses were considered exploratory and were not intended to compare the effectiveness or superiority of individual diet types. As an additional exploratory analysis, overall staple-food and side-dish intake rates were examined separately and simultaneously using multivariable ordinal logistic regression adjusted for the same covariates as the primary models. The correlation between staple-food and side-dish intake rates was assessed using Pearson’s correlation coefficient. For the exploratory intake-rate analyses, effect sizes were reported as ORs per 10% increase in the corresponding intake rate with 95% confidence intervals (CIs). All statistical tests were two-sided, and a *p*-value < 0.05 was considered statistically significant. All analyses were performed using IBM SPSS Statistics (version 29.0.0.0; IBM Corp., Armonk, NY, USA).

## 3. Results

### 3.1. Patient Characteristics

A total of 450 patients with acute stroke who were prescribed TMDs during hospitalization were initially identified. After excluding patients with missing data for key variables and those who received TMDs for fewer than 5 consecutive days, 406 patients were included in the final analysis (Figure 1). The demographic and clinical characteristics of the participants are summarized in Table 1. The median age was 81 years, and 194 patients (47.8%) were women. Ischemic stroke and intracerebral hemorrhage were observed in 271 (66.7%) and 135 (33.3%) patients, respectively. The median NIHSS score at admission was 14, indicating that the study population included predominantly patients with moderate-to-severe stroke. The median mRS score was 4 [IQR, 4–4] at discharge and 4 [IQR, 3–5] at 90 days after stroke. Favorable functional outcomes, defined as an mRS score of 0–2, were observed in 32 patients (7.9%) at discharge and 63 patients (15.5%) at 90 days. Death within 90 days occurred in 20 patients (4.9%). The overall TMD intake rate and diet-specific intake rates are presented in Table 2.

### 3.2. Distribution of TMD Types and Intake Rates

Table 2 summarizes the distribution of TMD types and diet-specific intake rates. The most frequently prescribed diet type was the paste diet, followed by the jelly, mousse, and moist minced diets. The median meal-level intake rate was 0.68 (IQR, 0.55–0.85) for the jelly diet, 0.81 (IQR, 0.64–0.95) for the paste diet, 0.88 (IQR, 0.62–0.98) for the mousse diet, and 0.82 (IQR, 0.56–0.97) for the moist minced diet. Staple-food and side-dish intake rates were generally comparable across all diet types. Because some patients received more than one type of TMD during hospitalization, the numbers of patients receiving each diet type were not mutually exclusive.

### 3.3. Association Between Overall Intake Rate and Functional Outcomes

In the ordinal logistic regression analysis adjusted for age, sex, admission NIHSS score, premorbid mRS score, stroke type, and admission GNRI, each 10% increase in the overall TMD intake rate was associated with lower cumulative odds of being in a higher (worse) mRS category at discharge (OR, 0.85; 95% CI, 0.77–0.94) and at 90 days (OR, 0.88; 95% CI, 0.80–0.96) (Figure 2). Threshold-specific sensitivity analyses at 90 days showed a consistent direction of association across all possible mRS cut points, although the magnitude and statistical significance varied by threshold.

In the secondary binary logistic analyses, each 10% increase in the overall TMD intake rate was associated with higher odds of a favorable outcome (mRS 0–2) at discharge (OR, 1.31; 95% CI, 1.03–1.66; *p* = 0.030). At 90 days, the association was in the same direction but did not reach statistical significance (OR, 1.15; 95% CI, 0.98–1.35; *p* = 0.081). The associations observed in the primary ordinal models remained essentially unchanged after additional adjustment for hypertension, dyslipidemia, diabetes mellitus, and atrial fibrillation.

### 3.4. Association Between Diet-Specific Intake Rates and Functional Outcomes

The associations between diet-specific intake rates and functional outcomes in the exploratory analyses are shown in Figure 3 and Figure 4. In multivariable ordinal logistic regression models adjusted for age, sex, admission NIHSS score, premorbid mRS score, stroke type, and admission GNRI, the associations varied according to diet type and outcome time point. At discharge, a higher intake rate of the moist minced diet was significantly associated with lower odds of being in a higher (worse) mRS category, whereas no statistically significant associations were observed for the other diet types. At 90 days, none of the diet-specific intake rates were significantly associated with mRS score. Because diet type was selected and modified according to swallowing status and overall clinical condition, these diet-specific analyses were considered exploratory and should not be interpreted as comparisons of the effectiveness or superiority of individual diet types.

As an additional exploratory analysis, we examined the associations between staple-food and side-dish intake rates and functional outcomes. When evaluated separately in multivariable models adjusted for the same covariates as the primary analysis, higher staple-food and side-dish intake rates were each associated with lower odds of being in a higher (worse) mRS category at discharge and at 90 days. However, staple-food and side-dish intake rates were strongly correlated (Pearson r = 0.945). When both intake measures were entered simultaneously into the same model, neither remained statistically significant at discharge (staple food: adjusted OR, 0.84; 95% CI, 0.63–1.10; *p* = 0.204; side dishes: adjusted OR, 1.03; 95% CI, 0.76–1.40; *p* = 0.836) or at 90 days (staple food: adjusted OR, 0.83; 95% CI, 0.64–1.07; *p* = 0.152; side dishes: adjusted OR, 1.07; 95% CI, 0.81–1.41; *p* = 0.625). These findings indicate that the independent contributions of staple-food and side-dish intake could not be distinguished in the present dataset.

## 4. Discussion

In this retrospective observational study of patients with acute stroke who received TMDs, we found that a higher TMD intake rate was associated with better functional outcomes at discharge and 90 days after stroke onset. These associations remained after adjustment for age, sex, admission NIHSS score, premorbid mRS score, stroke type, and admission GNRI. Importantly, the present study evaluated not only whether a TMD was prescribed, but also the extent to which the prescribed diet was actually consumed.

Post-stroke dysphagia is a common and clinically important complication that has been associated with adverse outcomes, including aspiration pneumonia, malnutrition, mortality, and poor functional recovery [3,4,6,17,18,19]. In clinical practice, dysphagia management primarily focuses on swallowing safety, aspiration prevention, and selection of an appropriate dietary texture. However, prescribing a safe diet does not necessarily ensure adequate nutritional intake because the intended energy and protein provision can be achieved only if the prescribed meals are consumed in sufficient amounts. This issue is particularly relevant in institutionalized populations, including stroke survivors, in whom texture-modified foods may be associated with lower intake, reduced acceptability, or differences in nutrient provision depending on preparation and diet type [11,20,21,22,23]. In this context, our findings emphasize the importance of monitoring actual TMD intake rather than relying solely on TMD prescriptions during acute hospitalization.

Stroke severity, as assessed using the NIHSS score on admission, is a well-established determinant of functional prognosis [1]. However, our findings suggest that quantitative information on oral intake may provide additional clinically relevant information even after accounting for baseline neurological severity. In patients with dysphagia, intake of thickened fluids and TMDs is often insufficient to meet estimated fluid requirements [13,24], and dysphagia has been associated with an increased risk of malnutrition, particularly during the subacute and rehabilitation phases [6]. Inadequate hydration may also be reflected by laboratory markers of hydration status in patients with post-stroke dysphagia [14]. Although the present study did not directly assess energy or protein intake, persistently inadequate oral intake could plausibly reduce tolerance to rehabilitation and contribute to delayed recovery. Consistent with this concept, poor nutritional status at admission has been independently associated with worse survival and functional outcomes after stroke [5], supporting the importance of early identification of patients at nutritional risk. Therefore, quantitative assessment of oral intake may help identify patients who require early nutritional and swallowing interventions.

Several mechanisms may explain the observed association between the TMD intake rate and functional outcomes. First, patients who consumed a larger proportion of the prescribed TMDs may also have achieved greater energy and protein intake during the acute phase of stroke. However, actual energy and protein intakes were not directly measured in the present study; therefore, this potential mechanism remains speculative. This possibility is broadly consistent with a study of geriatric convalescent inpatients, in which patients with high adherence to a personalized diet demonstrated significantly greater improvement in motor Functional Independence Measure (FIM) scores over 8 weeks than those with low adherence, even after adjustment for baseline status and nutritional intake [25]. Second, adequate oral intake may facilitate participation in rehabilitation by maintaining physical endurance, alertness, and overall medical stability. Patients who consume a greater proportion of their prescribed meals may therefore tolerate rehabilitation and other aspects of acute stroke care better. This interpretation is broadly consistent with previous evidence showing that individualized nutritional support after stroke improves nutritional status, physical function, and dysphagia [26], although this mechanism has not been directly examined. Third, the TMD intake rate may also reflect swallowing ability and muscle-related vulnerability. Reduced intake after stroke may result not only from impaired swallowing safety but also from oral phase dysfunction, reduced tongue pressure, sarcopenia, fatigue, impaired consciousness, cognitive dysfunction, and poor appetite. This interpretation is supported by evidence that progression to more advanced food textures, reflecting improved swallowing function, is independently associated with greater FIM gain among older rehabilitation patients [27]. Furthermore, pre-existing sarcopenia in patients with acute stroke has been associated with adverse swallowing outcomes, suggesting that reduced oral intake may reflect broader impairment in swallowing and skeletal muscle function [16,28]. Finally, oral intake may also reflect overall recovery status, including consciousness, cognitive function, trunk control, appetite, and swallowing coordination. Therefore, the TMD intake rate should be interpreted not solely as a nutritional variable but as an integrated clinical indicator reflecting meal consumption, swallowing ability, overall medical condition, and readiness for rehabilitation. Because this study was observational, we could not determine whether a higher TMD intake rate directly improved functional recovery or whether patients with better recovery potential were simply able to consume more of their meal. Prospective studies incorporating detailed nutritional assessments, rehabilitation intensity, and serial swallowing evaluations are warranted to clarify the direction and mechanisms underlying this association.

In the exploratory diet-specific analyses, associations were not consistent across diet types or assessment time points. After multivariable adjustment, a statistically significant association was observed only for the moist minced diet at discharge, whereas no diet-specific association reached statistical significance at 90 days. These findings should be interpreted cautiously because diet types were selected and modified according to swallowing status and overall clinical condition, and some patients received more than one diet type during hospitalization. Thus, these analyses are susceptible to confounding by indication and should not be interpreted as demonstrating superiority of any particular diet type. Instead, the ability to consume the prescribed TMD may be a more clinically meaningful indicator of the patient’s clinical status and recovery potential. Although texture modification remains an essential strategy for improving swallowing safety in patients with dysphagia, it also presents a clinical dilemma because safer textures do not necessarily translate into adequate nutritional intake. Given the exploratory nature of these analyses and the absence of adjustment for multiple comparisons, the isolated association observed for the moist minced diet at discharge should therefore be interpreted cautiously.

For instance, thicker liquid consistencies may reduce immediate aspiration during instrumental assessment but may also increase pharyngeal residue or reduce fluid intake [9,29]. Conversely, when appropriately implemented, TMDs may improve energy and protein intake in patients with dysphagia [10,22]. Collectively, these findings highlight the importance of evaluating the appropriateness of the prescribed texture and the actual amount consumed. Therefore, management of post-stroke dysphagia should consider not only swallowing safety but also nutritional adequacy.

Previous studies have also suggested that not only the overall quantity but also different components of oral intake may be associated with functional recovery. Siotto et al. demonstrated that, among several food domains assessed using the plate waste method, waste of the “second dish” (a protein-rich course) was independently associated with activities of daily living recovery even after adjusting for age and serum albumin, whereas other food domains showed no independent association [15]. In the present study, when evaluated separately in multivariable models adjusted for the same covariates as the primary analysis, higher staple-food and side-dish intake rates were each associated with lower odds of worse mRS categories at discharge and at 90 days. However, when both intake measures were entered simultaneously into the same model, neither remained statistically significant. Given the strong correlation between staple-food and side-dish intake rates (Pearson r = 0.945), their independent contributions to functional outcomes could not be distinguished in the present dataset. Although we were unable to identify the independent contribution of a specific food component, as reported by Siotto et al. for protein-rich intake, our findings are broadly consistent with the concept that different components of oral intake, in addition to overall intake quantity, may be relevant to functional outcomes after stroke. Future studies incorporating nutrient-specific intake data, such as protein and energy intake, may help identify the dietary components most strongly associated with functional recovery during the acute phase.

This study has several clinical implications. First, the TMD intake rate may serve as a simple and readily available clinical indicator associated with functional outcomes in patients with acute stroke receiving TMDs for suspected or confirmed dysphagia. Second, patients with persistently low intake rates despite receiving TMDs may require more intensive assessment and intervention, including reassessment of diet texture, mealtime assistance, swallowing therapy, oral care, nutritional supplementation, and consideration of enteral nutrition when oral intake is insufficient [2,7,26,30,31,32]. Third, quantitative monitoring of oral intake may facilitate multidisciplinary decision-making among physicians, nurses, dietitians, speech-language therapists, and rehabilitation professionals. In particular, serial assessment of the TMD intake rate may help clinicians identify patients whose oral intake deteriorates or fails to improve despite texture modification.

The findings of this study should be interpreted in the context of contemporary stroke nutrition practices. Current stroke care emphasizes early enteral nutrition when safe and adequate oral intake cannot be achieved. The FOOD trial demonstrated the clinical importance of enteral feeding strategies in patients with dysphagic stroke [32], and current guidelines generally recommend early nasogastric feeding for patients with dysphagia who are unable to maintain adequate oral intake [2,32]. Furthermore, recent evidence suggests that frequent, individualized nutritional support may improve nutritional status, activities of daily living, and dysphagia rehabilitation outcomes after stroke [26]. Therefore, the present cohort, which was derived from 2012 to 2017, may not fully reflect current clinical practice, in which patients with insufficient oral intake may transition earlier to enteral nutrition, and the duration of TMD use may consequently be shorter. Nevertheless, this historical context provided a unique opportunity to examine the relationship between TMD intake and functional outcomes among patients who continued oral feeding during acute hospitalization.

This study has several limitations. First, this was a single-center retrospective observational study, and causality cannot be inferred. Diet texture was dynamically adjusted during hospitalization according to swallowing status and clinical course, and TMD intake was assessed only during periods in which TMDs were provided. Although the TMD intake rate was calculated as a mean proportion and was therefore not directly determined by the number of meals provided, differences in the duration of TMD provision and the number of recorded meals may have affected the precision of the intake estimate. Moreover, patients with greater recovery potential or more rapid improvement in swallowing, neurological status, or general clinical condition may have consumed a larger proportion of TMDs and subsequently progressed to less restrictive diets. Thus, reverse causality cannot be excluded. Second, although the primary models were adjusted for age, sex, admission NIHSS score, premorbid mRS score, stroke type, and admission GNRI, and sensitivity analyses additionally accounted for major vascular comorbidities, residual confounding remains possible. Factors such as cognitive function, level of consciousness, appetite, a history of eating disorders or other eating-related conditions, fatigue, oral hygiene, sarcopenia, dysphagia severity, and rehabilitation intensity were not systematically available and may have influenced both TMD intake and functional outcomes. In particular, although swallowing function was clinically assessed using the MWST, tolerance during oral intake, and speech-language-hearing therapist assessment when indicated, a standardized quantitative measure of dysphagia severity was not systematically recorded and could not be included as a covariate. Because diet selection and modification were also influenced by swallowing status and overall clinical condition, confounding by indication may remain, particularly in the exploratory diet-specific analyses. Third, TMD intake was visually estimated by nursing staff rather than measured by weighing the food consumed. Although intake was systematically recorded after each meal using the same clinical scale and separately for staple food and side dishes, visual estimation may have introduced measurement error and interobserver variability. Fourth, total energy, protein, and fluid intake were not directly measured. Therefore, the TMD intake rate should be interpreted as a measure of the proportion of prescribed TMDs consumed rather than as a direct measure of nutrient intake. This limits our ability to determine the nutritional mechanisms underlying the observed associations. Fifth, respiratory infections, including upper respiratory tract infections and pneumonia, were not systematically captured using predefined diagnostic criteria. Their incidence and associations with TMD intake and functional outcomes therefore could not be reliably evaluated, and the potential influence of respiratory complications on oral intake and subsequent outcomes cannot be excluded. Finally, selection bias related to the requirement for at least 5 consecutive days of TMD consumption should be considered. Patients with very short periods of TMD use, including those who rapidly transitioned to tube feeding or to less restrictive diets, were excluded from the analysis. Therefore, the present findings are most applicable to patients who received TMDs for at least 5 consecutive days during hospitalization.

## 5. Conclusions

A higher TMD intake rate was associated with better functional outcomes at discharge and 90 days after stroke onset in patients with acute stroke who received TMDs for suspected or confirmed dysphagia. These findings suggest that monitoring the actual amount of TMD consumed, rather than the TMD prescription alone, may provide clinically useful information about patients’ oral intake status and may help prompt closer nutritional and swallowing assessment. Because this was an observational study, further prospective studies are needed to clarify whether interventions aimed at improving TMD intake can contribute to functional recovery after stroke.

## Figures and Tables

**Figure 1:**
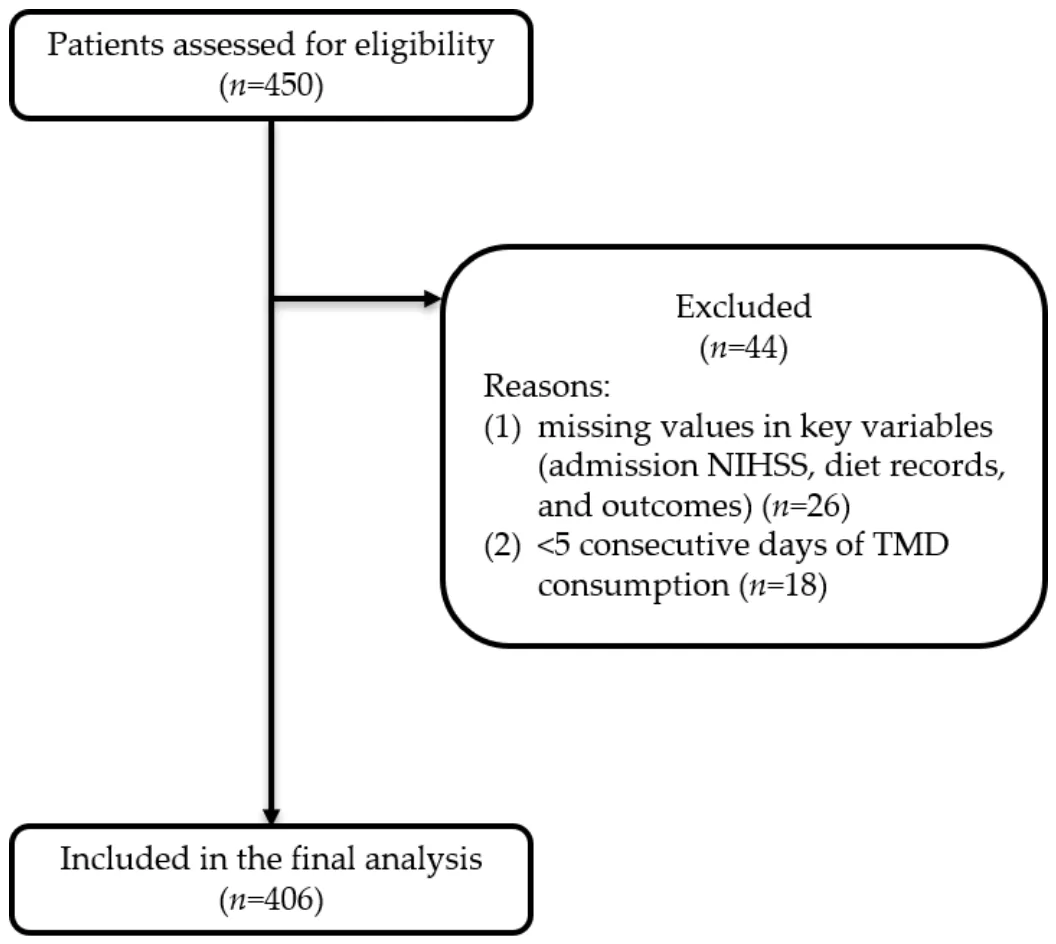
Flow diagram of patient selection. A total of 450 patients with acute stroke who were prescribed texture-modified diets (TMDs) during hospitalization were assessed for eligibility. After excluding 44 patients because of missing data for key variables or fewer than 5 consecutive days of TMD consumption, 406 patients were included in the final analysis. NIHSS, National Institutes of Health Stroke Scale; TMDs, texture-modified diets.

**Figure 2:**
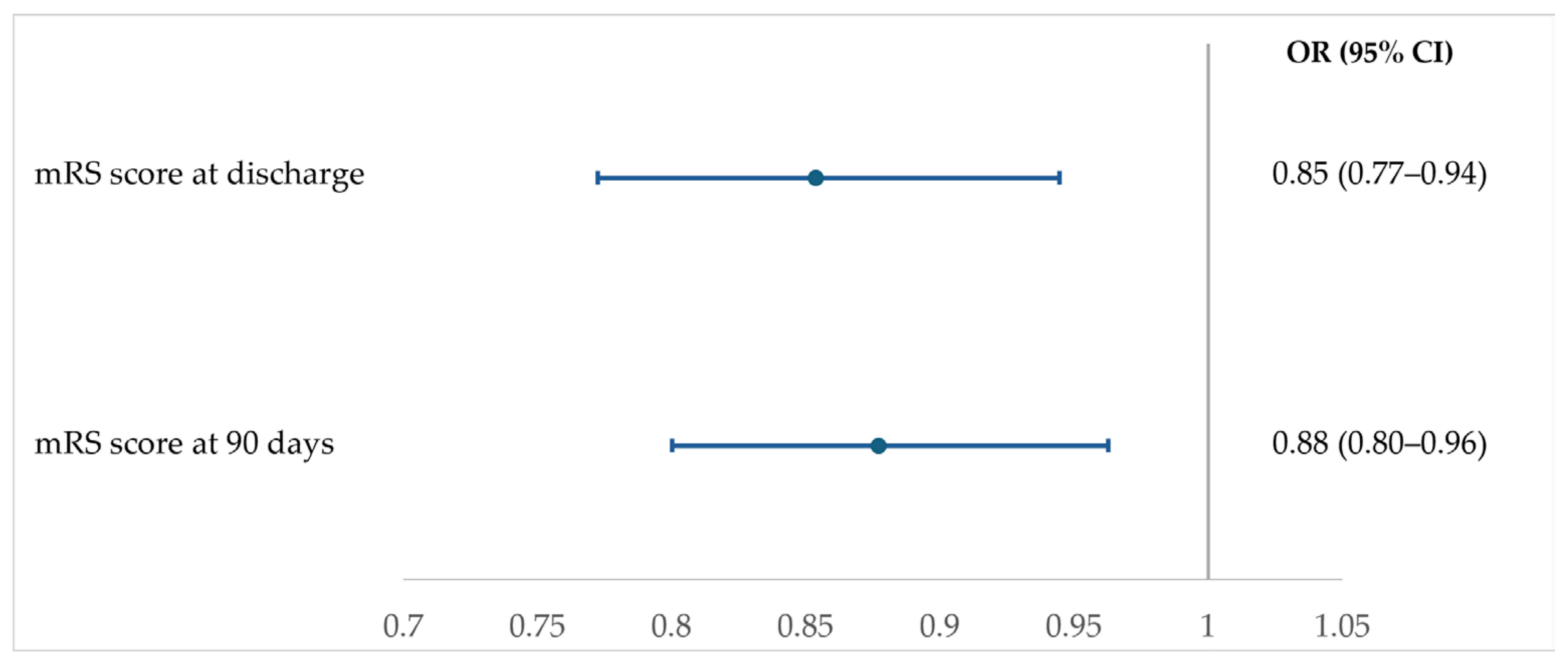
Association between the overall texture-modified diet (TMD) intake rate and modified Rankin Scale (mRS) scores at discharge and 90 days after stroke. Odds ratios (ORs) are presented per 10% increase in the overall TMD intake rate and are adjusted for age, sex, admission National Institutes of Health Stroke Scale (NIHSS) score, premorbid mRS score, stroke type, and admission Geriatric Nutritional Risk Index (GNRI). An OR < 1 indicates lower odds of being in a higher (worse) mRS category.

**Figure 3:**
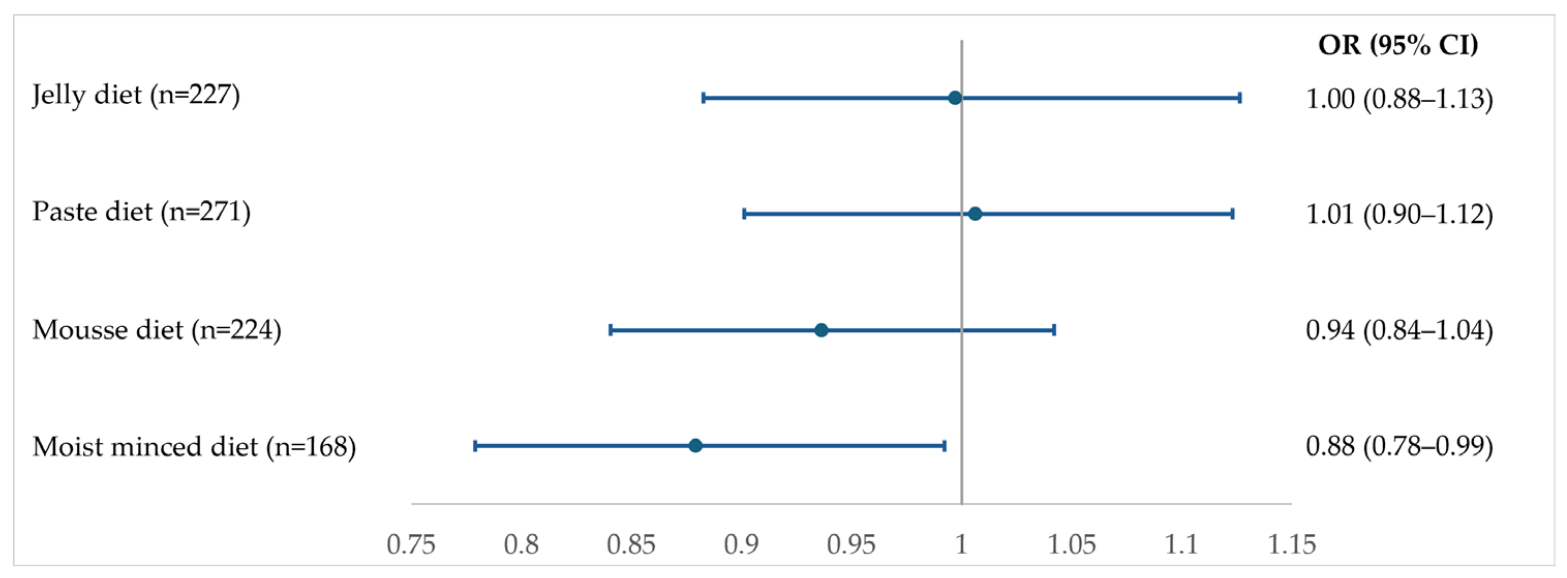
Exploratory association between diet-specific texture-modified diet (TMD) intake rates and modified Rankin Scale (mRS) scores at discharge. Odds ratios are presented per 10% increase in the corresponding diet-specific intake rate and are adjusted for age, sex, admission NIHSS score, premorbid mRS score, stroke type, and admission GNRI. Diet-specific analyses were exploratory and should not be interpreted as evidence of superiority of any specific TMD type. Because some patients received more than one TMD type during hospitalization, the diet-specific groups were not mutually exclusive.

**Figure 4:**
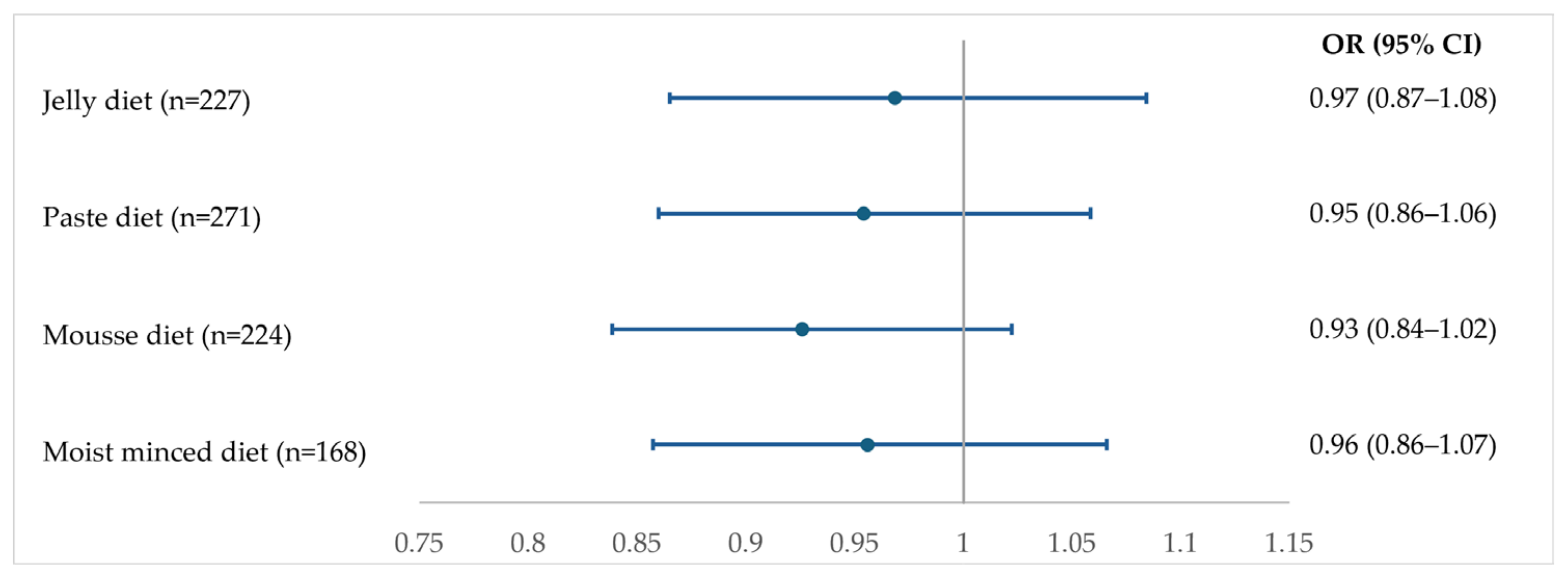
Exploratory association between diet-specific texture-modified diet (TMD) intake rates and modified Rankin Scale (mRS) scores at 90 days. Odds ratios are presented per 10% increase in the corresponding diet-specific intake rate and are adjusted for age, sex, admission NIHSS score, premorbid mRS score, stroke type, and admission GNRI. Diet-specific analyses were exploratory and should not be interpreted as evidence of superiority of any specific TMD type. Because some patients received more than one TMD type during hospitalization, the diet-specific groups were not mutually exclusive.

**Table 1:** Demographic and clinical characteristics of the study participants.

Characteristic	Overall Cohort (*n* = 406)
Age, years	81 [72–86]
Female sex	194 (47.8)
Stroke type	
Ischemic stroke	271 (66.7)
Intracerebral hemorrhage	135 (33.3)
NIHSS score at admission	14 [7–21]
Premorbid mRS score	1 [0–3]
mRS 0–2	255 (62.8)
mRS 3–5	151 (37.2)
Hypertension	321 (79.1)
Dyslipidemia	159 (39.2)
Diabetes mellitus	88 (21.7)
Atrial fibrillation	137 (33.7)
Admission GNRI	98.1 [90.9–105.5]
Length of hospital stay, days	24 [18–31]
Number of TMD meals	23 [20–25]
mRS score at discharge	4 [4–4]
mRS 0–2	32 (7.9)
mRS 3–5	374 (92.1)
mRS score at 90 days	4 [3–5]
mRS 0–2	63 (15.5)
mRS 3–5	323 (79.6)
mRS 6 (death)	20 (4.9)

Data are presented as median [interquartile range] or number (%). GNRI, Geriatric Nutritional Risk Index; NIHSS, National Institutes of Health Stroke Scale; mRS, modified Rankin Scale.

**Table 2:** Distribution of texture-modified diet types and intake rates.

Diet Type	Patients Receiving This Diet, *n* (%)	Meals Served, *n*	Meals per Patient, Median [IQR]	Staple-Food Intake Rate, Median [IQR]	Side-Dish Intake Rate, Median [IQR]	Meal-Level Intake Rate, Median [IQR]
Overall TMD intake	406 (100.0)	8896	23.0 [20.0–25.0]	0.78 [0.60–0.92]	0.79 [0.66–0.92]	0.78 [0.64–0.92]
Jelly diet	227 (55.9)	1991	7.0 [4.0–12.0]	0.67 [0.49–0.85]	0.70 [0.57–0.86]	0.68 [0.55–0.85]
Paste diet	271 (66.7)	2719	9.0 [5.0–14.0]	0.80 [0.60–0.94]	0.83 [0.68–0.95]	0.81 [0.64–0.95]
Mousse diet	224 (55.2)	2133	8.0 [4.0–14.0]	0.88 [0.62–0.99]	0.87 [0.63–0.98]	0.88 [0.62–0.98]
Moist minced diet	168 (41.4)	2053	10.0 [5.0–21.0]	0.84 [0.53–0.99]	0.83 [0.55–0.97]	0.82 [0.56–0.97]

Values are presented as *n* (%) or median [interquartile range]. Intake rates ranged from 0 to 1, with 0 indicating no intake and 1 indicating complete consumption of the provided meal component. The meal-level intake rate was calculated as the mean of the staple-food and side-dish intake rates. Diet-type-specific medians and interquartile ranges were calculated at the patient level among patients who received each diet type. Because some patients received more than one diet type during hospitalization, the numbers of patients across diet types are not mutually exclusive and may exceed the total number of patients. The overall TMD intake rate was calculated across all TMD meals provided during the study period. IQR, interquartile range; TMD, texture-modified diet.

## Data Availability

The data supporting the findings of this study are available from the corresponding author upon reasonable request. The data are not publicly available because of privacy and ethical restrictions.

## Supplementary Materials

The following supporting information can be downloaded at: https://www.mdpi.com/article/10.3390/nu18193114/s1, Figure S1: Representative photographs of texture-modified diets corresponding to the diet categories examined in this study.

## Abbreviations

The following abbreviations are used in this manuscript:

mRS	Modified Rankin Scale
NIHSS	National Institutes of Health Stroke Scale
TMD	Texture-modified diet
GNRI	Geriatric Nutritional Risk Index
MWST	Modified Water Swallowing Test
IDDSI	International Dysphagia Diet Standardisation Initiative
FIM	Functional Independence Measure
FOIS	Functional Oral Intake Scale
