# Supplementary material for: Association Between Texture-Modified Diet Intake Rate and Functional Outcomes After Acute Stroke: A Retrospective Observational Study
Source: Nutrients. 2026 Sep 22;18(19):3114. doi: 10.3390/nu18193114 (PMC13667115; doi:10.3390/nu18193114)
Supplement: Supplementary file 1 [file nutrients-18-03114-s001.zip › nutrients-4486590-supplementary.pdf]

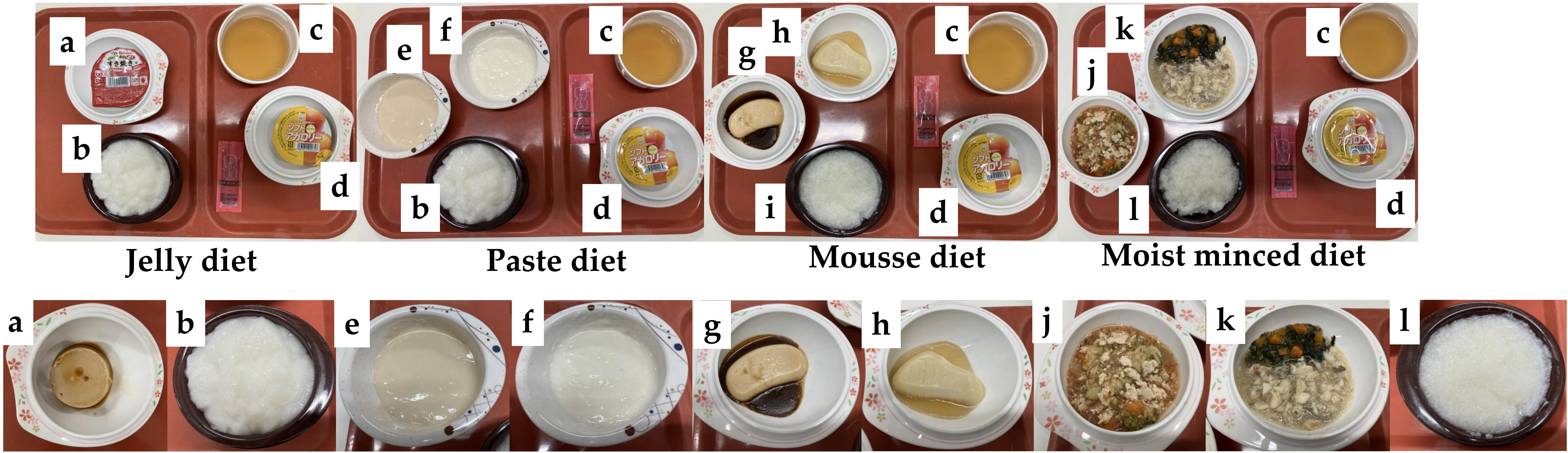

Supplementary Figure S1. Representative photographs of texture-modified diets corresponding to the diet categories examined in this study.
Representative examples of the four texture-modified diet categories are shown: jelly diet, paste diet, mousse diet, and moist minced diet. The individual items are as follows: (a) sukiyaki jelly; (b) blended rice porridge; (c) tea jelly; (d) high-calorie cup jelly; (e) pork paste; (f) white fish paste; (g) pork mousse served with stew sauce; (h) white fish mousse served with thickened Chinese-style sauce; (i) mashed rice porridge; (j) chicken in tomato sauce; (k) simmered red fish; and (l) Full rice porridge. These photographs show examples of texture-modified diets currently provided at our institution to illustrate the representative appearance of each diet category. They are not identical to the meals served during the 2012–2017 study period and do not represent fixed menus provided to all patients.
